# Author Correction: Mechanically active integrins target lytic secretion at the immune synapse to facilitate cellular cytotoxicity

**DOI:** 10.1038/s41467-023-44258-z

**Published:** 2023-12-18

**Authors:** Mitchell S. Wang, Yuesong Hu, Elisa E. Sanchez, Xihe Xie, Nathan H. Roy, Miguel de Jesus, Benjamin Y. Winer, Elizabeth A. Zale, Weiyang Jin, Chirag Sachar, Joanne H. Lee, Yeonsun Hong, Minsoo Kim, Lance C. Kam, Khalid Salaita, Morgan Huse

**Affiliations:** 1 Immunology Program, Memorial Sloan Kettering Cancer Center, New York, NY USA; 2 Pharmacology Program, Weill-Cornell Graduate School of Medical Sciences, New York, NY USA; 3 Department of Chemistry, Emory University, Atlanta, GA USA; 4 Biochemistry and Molecular Biology Program, Weill-Cornell Graduate School of Medical Sciences, New York, NY USA; 5 Neuroscience Program, Weill-Cornell Graduate School of Medical Sciences, New York, NY USA; 6 Department of Microbiology and Immunology, State University of New York Upstate Medical University, Syracuse, NY USA; 7 Department of Biomedical Engineering, Columbia University, New York, NY USA; 8 Department of Microbiology and Immunology, University of Rochester Medical Center, Rochester, NY USA

**Keywords:** Cytotoxic T cells, Integrins, Imaging the immune system, Signal transduction

Correction to: *Nature Communications* 10.1038/s41467-022-30809-3, published online 09 June 2022

The original version of this Article contained two errors. In Fig. 8f/8g, the in-figure legend incorrectly spelled “NTCR+P/I” and “TlnCR+P/I”, while they should spell “NTCR+Tln-head” and TlnCR+Tln-head”, respectively. In Supplementary Fig. 10d, the four in-figure legends spelled “NTCR”, “TlnCR”, “NTCR+Tln-head” and “TlnCR+Tln-head”, while they should spell “NTCR”, “NTCR+Tln-head”, “TlnCR” and “TlnCR+Tln-head”, respectively. These errors have now been corrected in both the PDF and HTML versions of the article.

### Supplementary information

Updated Supplementary Information

