# Supplementary material for: Mechanically active integrins target lytic secretion at the immune synapse to facilitate cellular cytotoxicity
Source: Nat Commun. 2022 Jun 9;13:3222. doi: 10.1038/s41467-022-30809-3 (PMC9184626; doi:10.1038/s41467-022-30809-3)
Supplement: Supplementary file 1 — Supplementary Information [file 41467_2022_30809_MOESM1_ESM.pdf]

Supplementary information for

Mechanically active integrins help target lytic secretion at the immune synapse to facilitate cellular cytotoxicity

Mitchell S. Wang, Yuesong Hu, Elisa E. Sanchez, Xihe Xie, Nathan H. Roy, Miguel de Jesus, Benjamin Y. Winer, Elizabeth A. Zale, Weiyang Jin, Chirag Sachar, Joanne H. Lee, Yeonsun Hong, Minsoo Kim, Lance C. Kam, Khalid Salaita, and Morgan Huse*

* correspondence: husem@mskcc.org

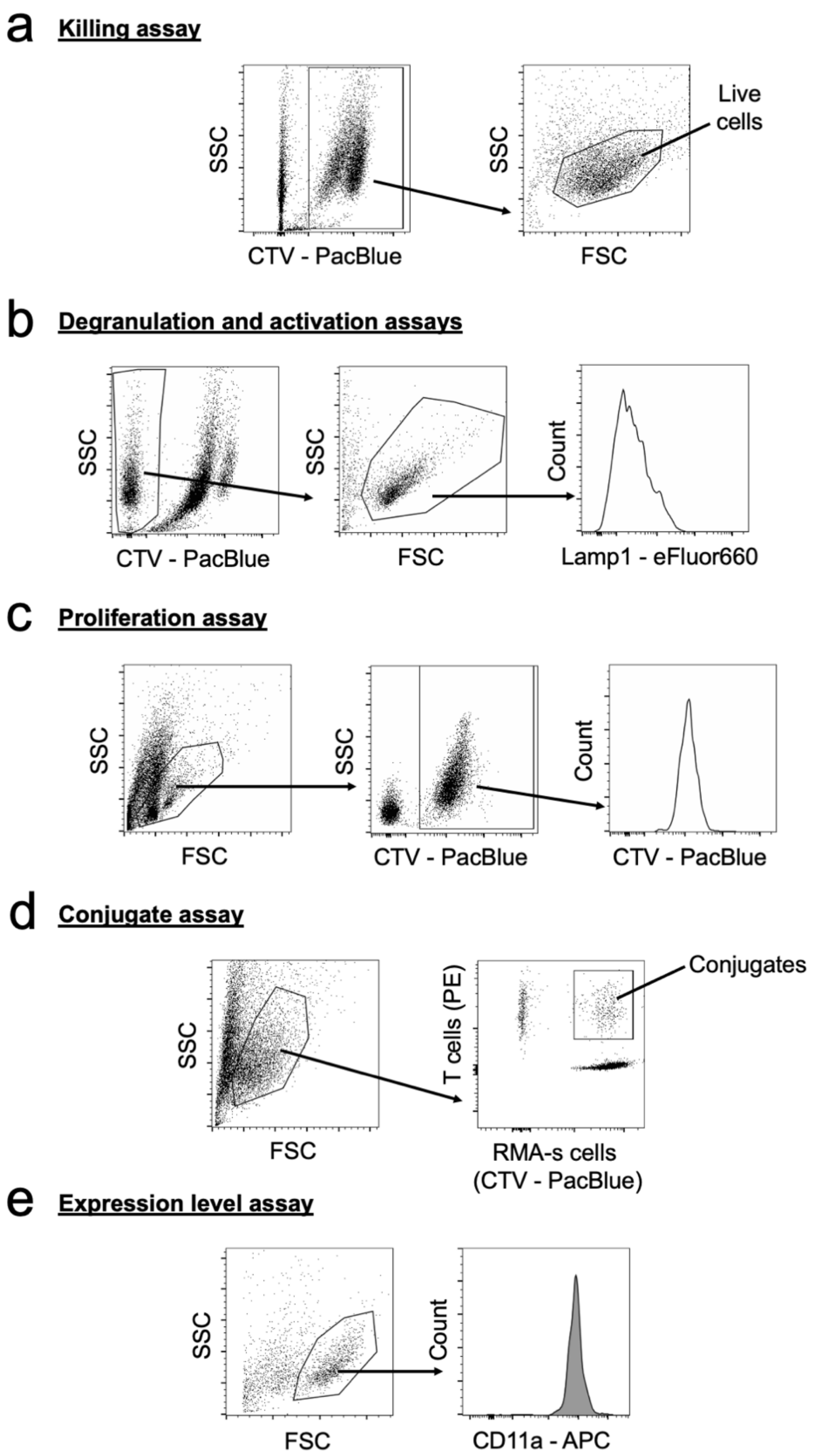

Supplementary Figure 1. Gating strategies used for analysis. Gating strategies used for in vitro co-culture assays. (a) Gating strategy used to measure T cell cytotoxicity against peptide-loaded RMA-s target cells (CellTrace Violet, CTV$^+$) via forward/side-scatter. (b) Gating strategy used to measure live T cell (CTV$^-$) degranulation (via Lamp1 exposure) and activation (via CD69 upregulation, not shown) against peptide-loaded RMA-s target cells (CTV$^+$). (c) Gating strategy

used to measure live T cell proliferation (CTV^+) via dilution of CTV dye over consecutive days. (d) Gating strategy used to measure live T cell conjugate formation against peptide-loaded RMA-s target cells (conjugates defined as post-fixation events double positive for CTV and PKH26 (visible in the PE channel). (e) Gating strategy used to measure surface expression of LFA1 on T cells stimulated with peptide and ICAM-1-coated beads (T cells identified via forward/side-scatter morphology).

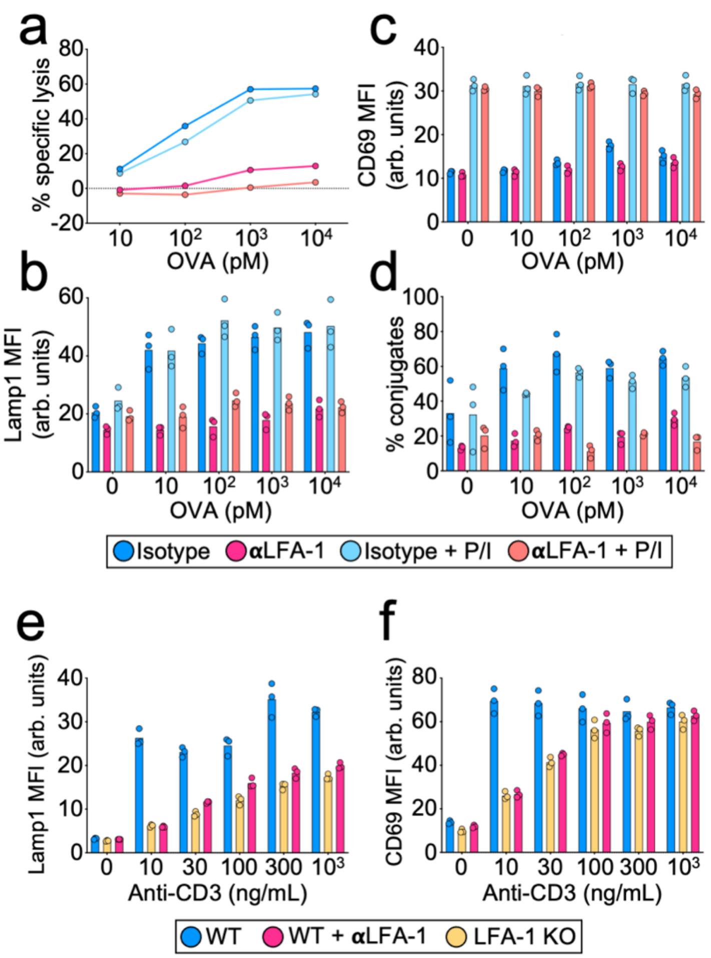

Supplementary Figure 2. LFA-1 is required for CTL degranulation and cytotoxicity. Replicate experiments for Fig. 2. (a-d) OVA-loaded RMA-s target cells were mixed with OT-1 CTLs in the presence of LFA1 blocking antibody (αLFA-1) or isotype control. PMA/Iono (P/I) was applied to some samples in order to drive TCR independent CTL activation. (a) Target cell killing, measured 4 h after CTL-target cell mixing (N = 2 replicate experiments). (b) Lamp1 exposure (degranulation), measured 90 min after CTL-target cell mixing (N = 4 replicate experiments). (c) CD69 expression, measured 90 min after CTL-target cell mixing (N = 2 replicate experiments). (d) Conjugate formation, measured 90 min after CTL-target cell mixing (N = 3 replicate experiments). (e-f) Polyclonal WT and LFA-1 KO CTLs were mixed with stimulatory beads coated with anti-CD3 antibody + ICAM-1 in the presence or absence of LFA1 blocking antibody (αLFA-1) as indicated. (e) Lamp1 exposure, measured 90 min after CTL-bead mixing (N = 2 replicate experiments). (f) CD69 expression, measured 90 min after CTL-bead mixing (N = 2 replicate experiments). Data points in b-f represent technical triplicate measurements from an individual experiment. In a, data points represent mean values calculated from technical triplicates. Source data are provided as a Source Data file.

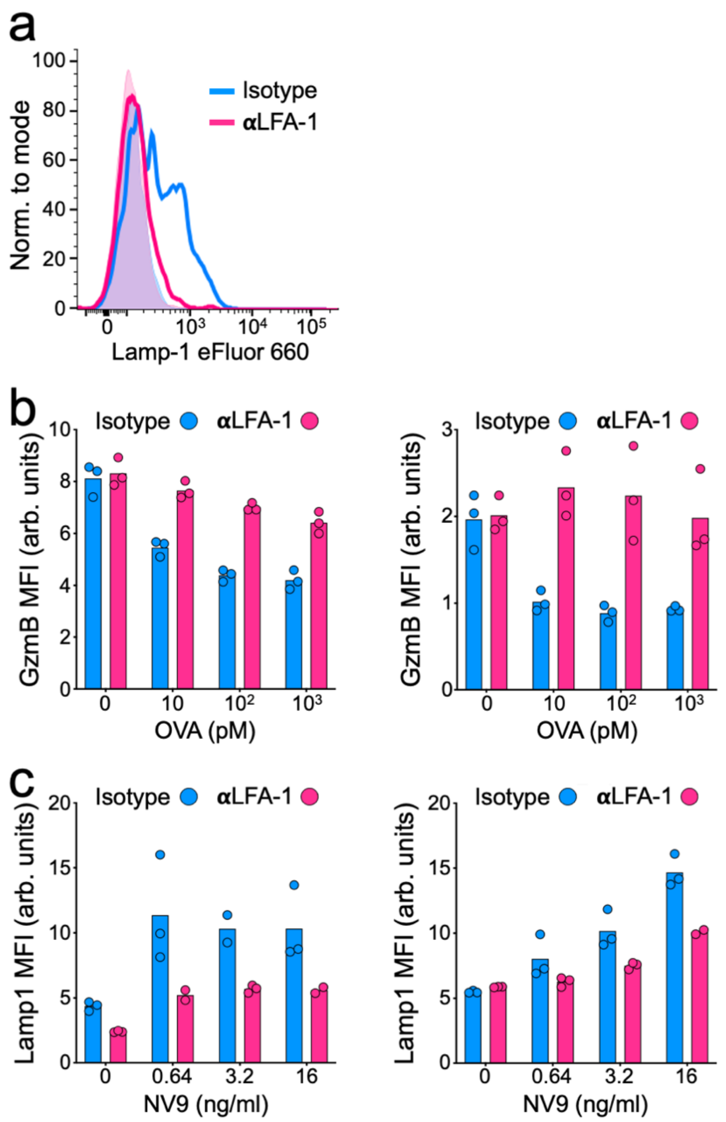

Supplementary Figure 3. LFA-1 blockade disrupts degranulation in CTL-target cell conjugates. (a-b) OT-1 CTLs were incubated with OVA-loaded RMA-s cells in the presence of LFA1 blocking antibody (αLFA-1) or isotype control. (a) A representative FACS histogram of CTL Lamp1 staining, measured 90 min after cell mixing. (b) Staining for intracellular granzyme B (GzmB) in CTLs, performed 90 min after cell mixing. N = 2 replicate experiments, which are both shown. (c) Human OM265 CMV CTLs were mixed with HLA-A2^{+} CD4^{+} T cell targets in the presence of αLFA-1 or isotype control. CTL Lamp1 exposure was measured after 4 h. N = 2 replicate experiments, which are both shown. Data points in b-c represent technical replicate measurements from individual experiments. Source data are provided as a Source Data file.

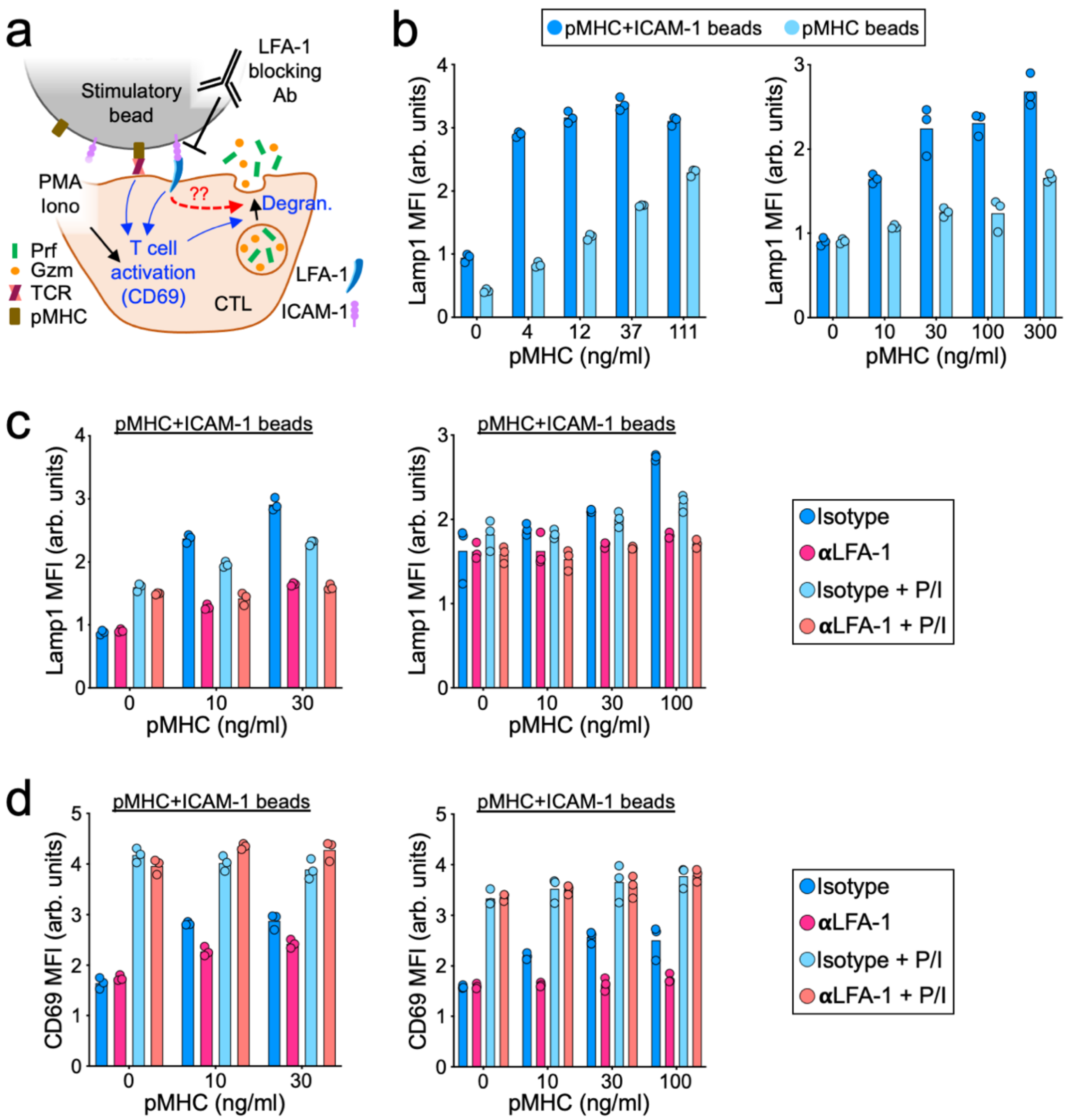

Supplementary Figure 4. LFA-1 is required for CTL degranulation responses to stimulatory beads. (a) Diagram schematizing CTL activation by stimulatory beads coated with pMHC (H-2K^{b}-OVA) ± ICAM-1. (b) Degranulation responses to beads coated with pMHC ± ICAM-1, measured 90 min after CTL stimulation. N = 5 replicate experiments, two of which are shown. (c-d) Beads coated with pMHC and ICAM-1 were incubated with OT-1 CTLs in the presence or absence of PMA/Iono and treated with either αLFA-1 or isotype control. Graphs show degranulation (c) and CD69 expression (d), measured 90 min after CTL stimulation. N = 2 replicate experiments, which are both shown. Data points in b-c represent technical replicate measurements from individual experiments. Source data are provided as a Source Data file.

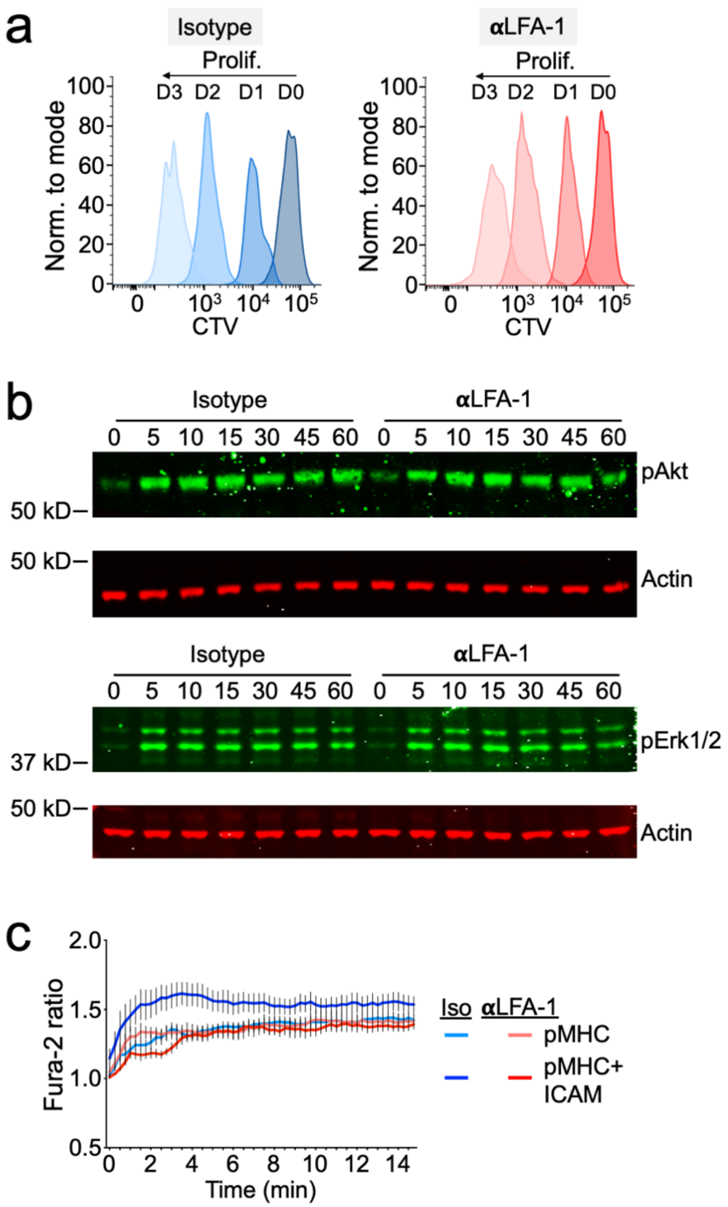

Supplementary Figure 5. LFA-1 blockade alters only some indices of T cell activation. (a) OT-1 CTLs were labeled with CellTrace Violet (CTV) and incubated with OVA-loaded splenocytes in the presence of αLFA-1 or isotype control. CTV dilution was monitored over 3 days (D) by flow cytometry. (b) OT-1 CTLs were mixed with pMHC (H-2K^b-OVA) and ICAM-1 coated beads and, at the indicated timepoints, pAKT (top) and pErk1/2 (bottom) were assessed by immunoblot, with actin serving as a loading control. Molecular weight standards are indicated to the left of each blot. (c) OT-1 CTLs were loaded with Fura-2-AM and imaged on glass surfaces coated with the indicated proteins in the presence of αLFA-1 or isotype control. Graph shows the mean Fura-2 ratio of all CTLs in the imaging field, averaged over 6 positions. Error bars signify SEM. All data

are representative of at least two independent experiments. Source data are provided as a Source Data file. Uncropped immunoblots are shown in Supplementary Fig. 12.

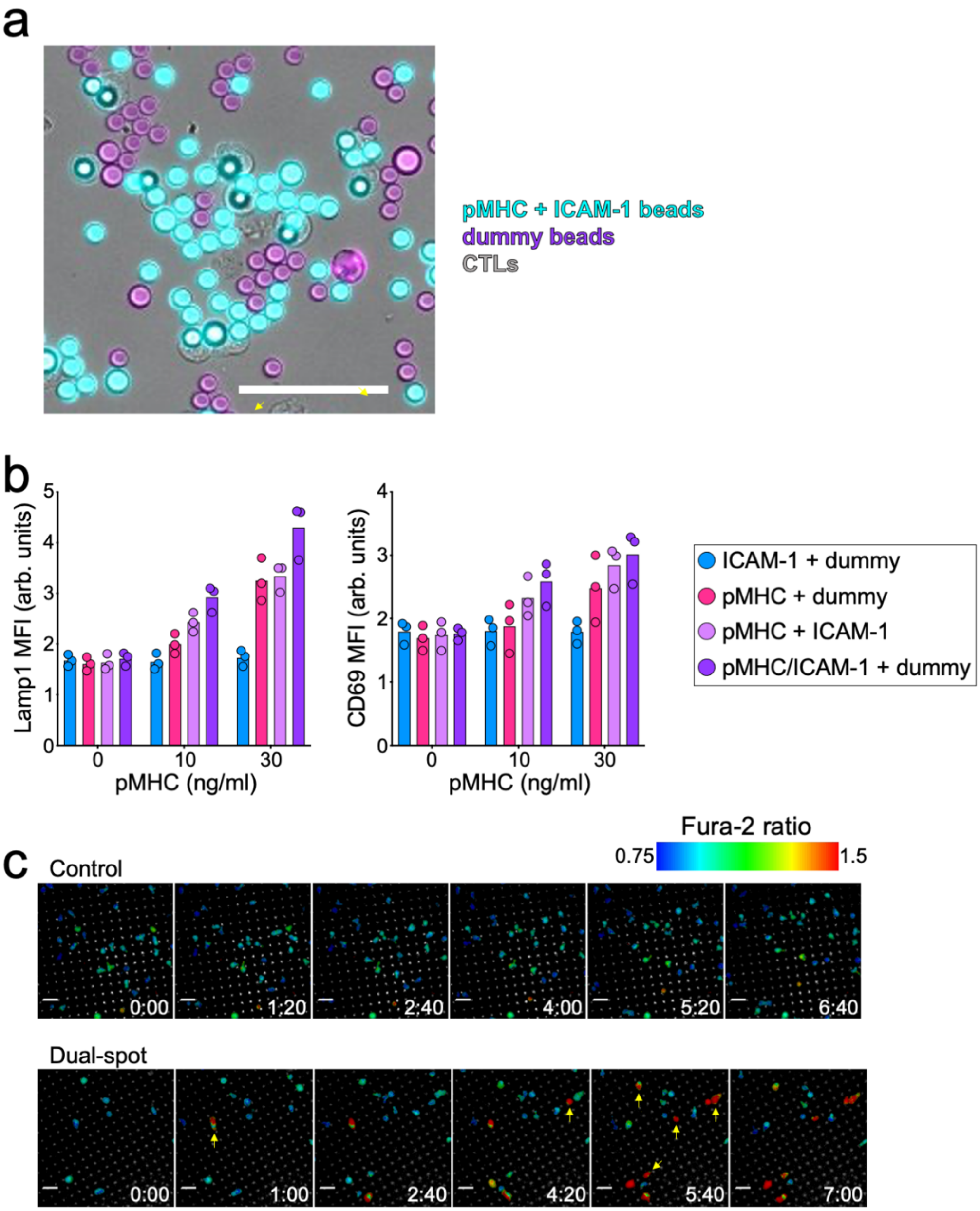

Supplementary Figure 6. CTL stimulation with spatially segregated ligands. (a) Representative image of OT-1 CTLs, beads coated with both pMHC and ICAM-1, and dummy beads mixed at the proportions used for Figure 3. Scale bar = 50 μm. (b) Replicate experiments for Fig. 3b-c. OT-1 CTLs were activated by equal mixtures of stimulatory beads bearing the indicated proteins. Graphs showing CTL degranulation (left) and CD69 expression (right), measured 90 min after CTL stimulation (N = 2 replicate experiments). Data points represent technical triplicate measurements from an individual experiment. Source data are provided as a Source Data file. (c) Representative time-lapse montages of Fura-2-AM-loaded OT-1 CTLs imaged on Dual-spot and control surfaces. Yellow arrows denote the onset of Ca^{2+} flux in

individual cells. Time in M:SS is shown in the bottom right corner of each image. Scale bars = 10 μm.

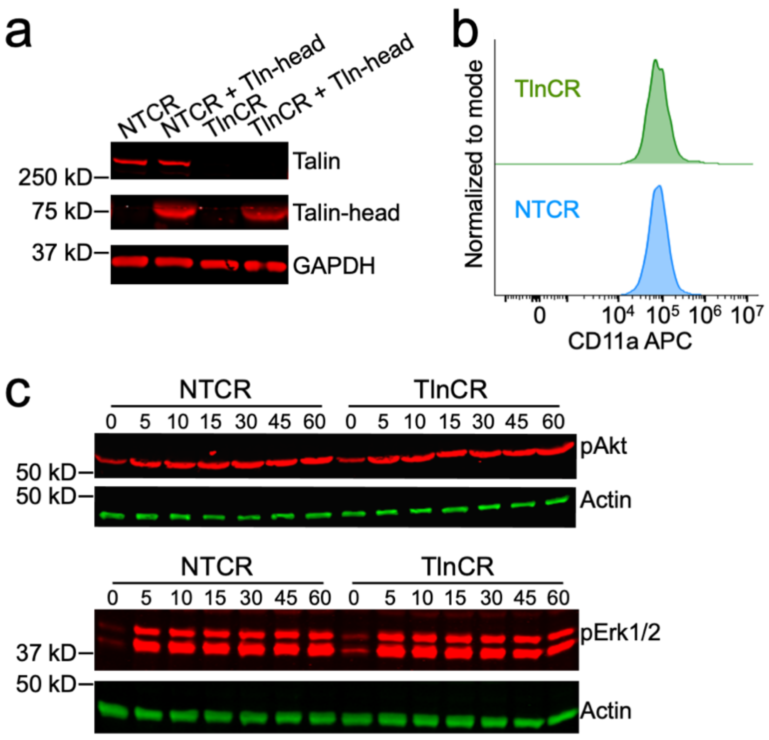

Supplementary Figure 7. Talin depletion inhibits degranulation but not early TCR signaling. (a) Immunoblot analysis of full-length talin and the GFP-talin head domain in OT-1 Cas9 CTLs transduced with the indicated gRNAs ± talin head. NTCR = nontargeting gRNA, TlnCR = talin gRNA. GAPDH served as a loading control. Molecular weight standards are indicated to the left of each blot. (b) Representative flow cytometry histograms showing LFA-1 expression (quantified using an antibody against CD11a) on NTCR and TlnCR CTLs. (c) OT-1 Cas9 CTLs transduced with the indicated gRNAs were mixed with pMHC (H-2K^b-OVA) and ICAM-1 coated beads and, at the indicated timepoints, pAKT (top) and pErk1/2 (bottom) were assessed by immunoblot. Actin served as a loading control. Molecular weight standards are indicated to the left of each blot. Data are representative of at least two independent experiments. Uncropped immunoblots are shown in Supplementary Fig. 12.

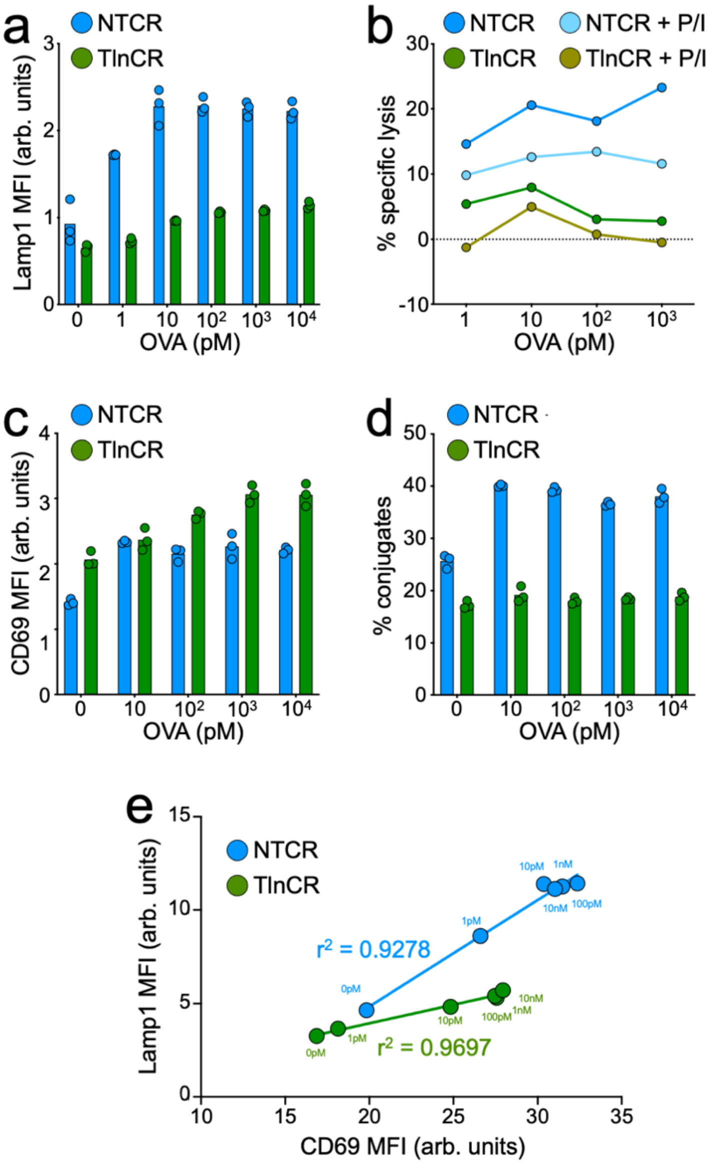

Supplementary Figure 8. Talin is required for CTL degranulation and cytotoxicity. (a-d) Replicate experiments for Fig. 7. OVA-loaded RMA-s target cells were mixed with OT-1 Cas9 CTLs expressing talin gRNA (TlnCR) or control nontargeting gRNA (NTCR). PMA/Iono (P/I) was applied to some samples in order to drive TCR independent CTL activation. (a) Lamp1 exposure (degranulation), measured 90 min after CTL-target cell mixing (N = 4 replicate experiments). (b) Target cell killing, measured 4 h after CTL-target cell mixing (N = 2 replicate experiments). (c) CD69 expression, measured 90 min after CTL-target cell mixing (N = 2 replicate experiments). (d) Conjugate formation, measured 90 min after CTL-target cell mixing (N = 2 replicate experiments). (e) 2-dimensional plot correlating degranulation (Lamp1) and T cell activation (CD69), taken from the same data set used to generate Fig. 7c. Regression lines and r^2 values are shown. Data points in a and c-d represent technical triplicate measurements from an individual

experiment. Data points in b and e represent mean values calculated from technical triplicates. Source data are provided as a Source Data file.

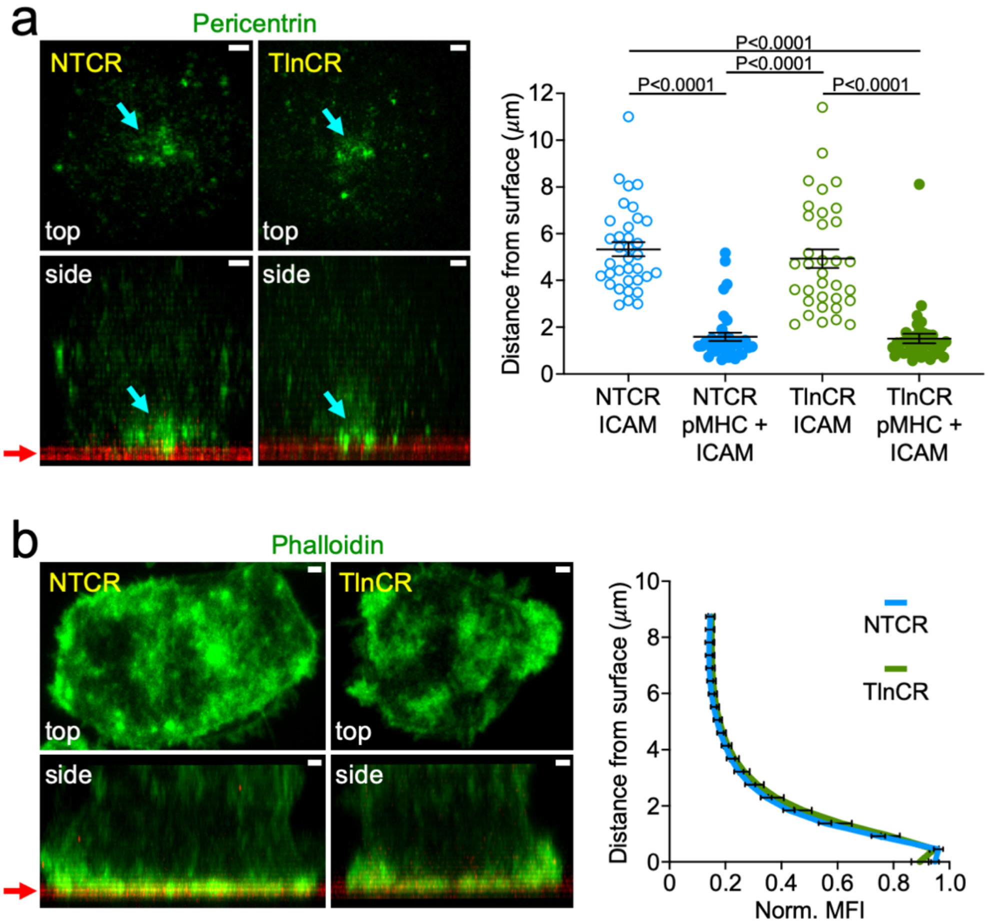

Supplementary Figure 9. Talin is dispensable for synaptic F-actin accumulation and centrosome polarization. OT-1 CTLs expressing the indicated gRNAs were fixed on pMHC + ICAM-1 coated coverslips and stained for pericentrin (a) or phalloidin (b). NTCR = nontargeting gRNA, TlnCR = talin gRNA. (a) Left, confocal images of representative cells, viewed from the top and side. In each set of images, the centrosome and the stimulatory surface are indicated by cyan and red arrows, respectively. Right, quantification of the distance between the centrosome and the surface. N ≥ 34 cells for each sample. P values were calculated by one-way ANOVA with Tukey correction. (b) Left, confocal images of representative cells, viewed from the top and side. The stimulatory surface is indicated by the red arrow in the side view images. Right, normalized mean phalloidin fluorescence intensity, graphed as a function of distance from the surface. Each curve was derived from ≥ 19 cells. All error bars signify SEM, and all scale bars = 1 μm. Data are representative of at least two independent experiments. Source data are provided as a Source Data file.

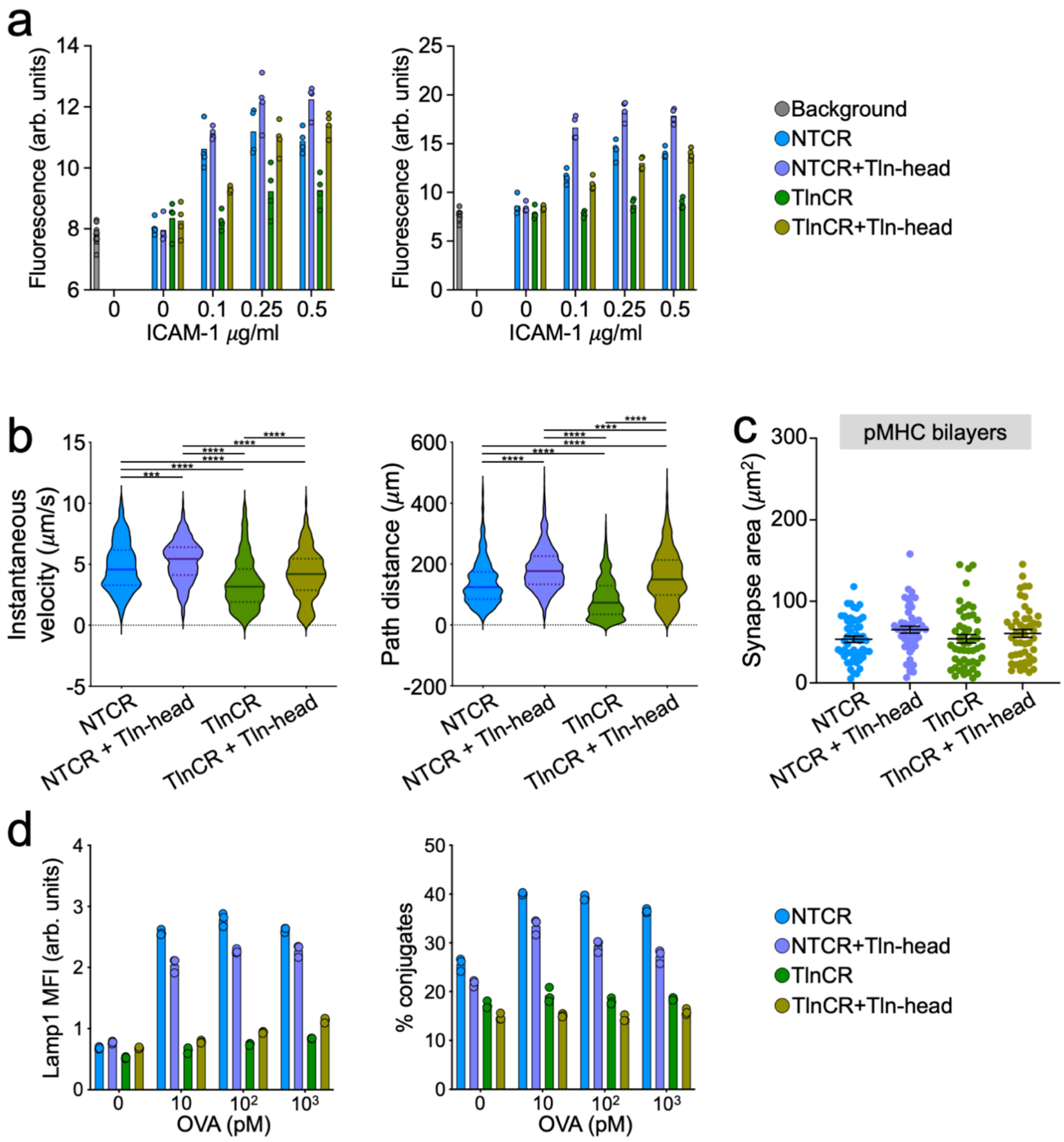

Supplementary Figure 10. The talin head domain rescues TlnCR CTL adhesion to and migration on ICAM-1, but not degranulation and conjugate formation. (a) OT-1 Cas9 CTLs transduced with talin gRNA (TlnCR) or control nontargeting gRNA (NTCR) ± the talin head domain (Tln-head) were applied to coverslips coated with increasing amounts of ICAM-1. Adhesion was measured after 20 min. Data points represent technical quadruplicate measurements from an individual experiment. Eight background readings are shown in gray. N = 2 replicate experiments, which are both shown. (b) OT-1 Cas9 CTLs transduced with the indicated gRNAs ± Tln-head were imaged on ICAM-1 coated coverslips. Instantaneous velocities (left) and total path distances (right) were derived from analysis of individual migratory tracks (N ≥ 414 tracks for each cell type). For each sample, solid lines denote medians and dotted lines indicate upper and lower quartiles.

Error bars signify SEM. *** and **** denote $P \leq 0.001$ and $P \leq 0.0001$, calculated by one-way ANOVA with Tukey correction. (c) OT-1 CTLs expressing the indicated gRNAs ± Tln-head were fixed on bilayers coated with pMHC alone and stained for phalloidin. IS area was quantified and graphed, with error bars denoting SEM. N ≥ 48 cells for each sample. Data in b and c are representative of two independent experiments. (d) Replicate experiments for Fig. 8f-g. OVA-loaded RMA-s target cells were mixed with OT-1 Cas9 CTLs expressing the indicated gRNAs ± Tln-head. Left, Lamp1 exposure (degranulation), measured 90 min after CTL-target cell mixing (N = 2 replicate experiments). Right, conjugate formation, measured 90 min after CTL-target cell mixing (N = 2 replicate experiments). Data points represent technical triplicate measurements from an individual experiment. Source data for all graphs are provided as a Source Data file.

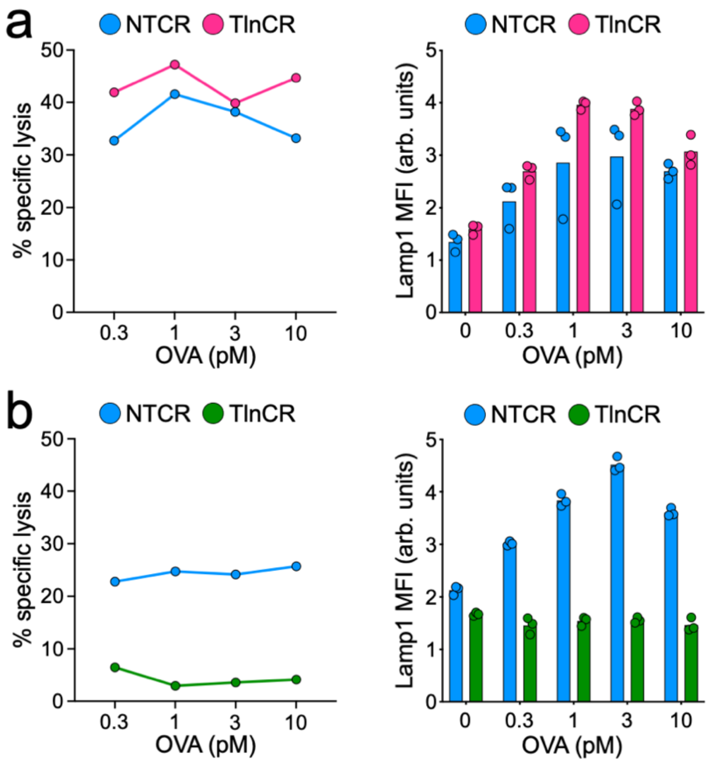

Supplementary Figure 11. Talin, but not LFA-1, is required for CTL-mediated killing of B16F10 cells. Replicate experiments for Fig. 9c-d. (a) OVA-loaded B16F10 target cells were mixed with OT-1 CTLs in the presence of LFA1 blocking antibody (αLFA-1) or isotype control. Left, specific lysis, measured after 4 h (N = 2 replicate experiments). Right, Lamp1 exposure, measured after 90 min (N = 2 replicate experiments). (b) OVA-loaded B16F10 target cells were mixed with OT-1 Cas9 CTLs transduced with talin gRNA (TlnCR) or control nontargeting gRNA (NTCR). Left, specific lysis, measured after 4 h (N = 2 replicate experiments). Right, Lamp1 exposure, measured after 90 min (N = 2 replicate experiments). Data points in degranulation graphs represent technical triplicate measurements from an individual experiment. Data points in killing assays represent mean values calculated from technical triplicates. Source data are provided as a Source Data file.

pAKT

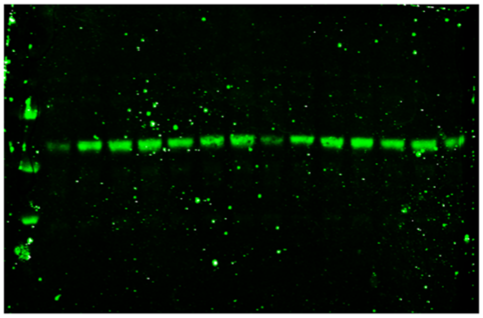

Actin

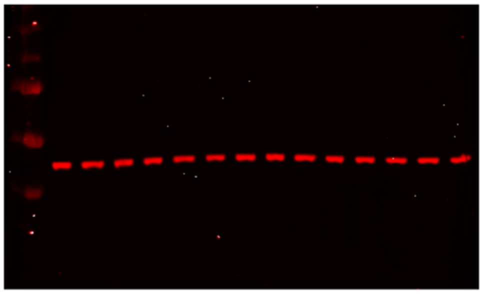

Supp. Fig. 5b

pErk1/2

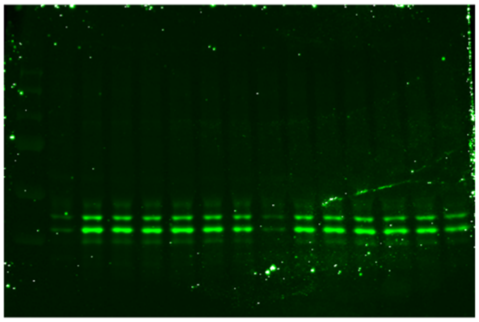

Actin

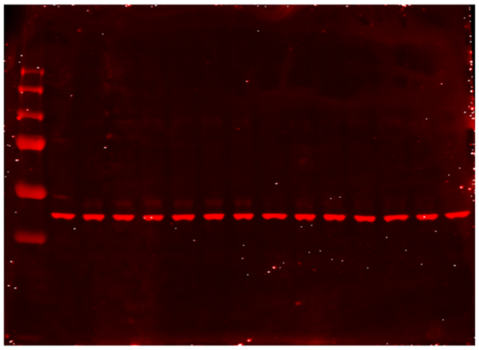

Talin

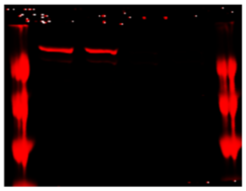

Sup. Fig. S7a

Talin head (GFP)

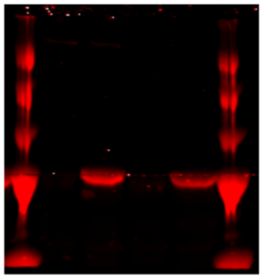

GAPDH

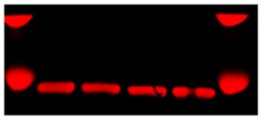

pAKT

Actin

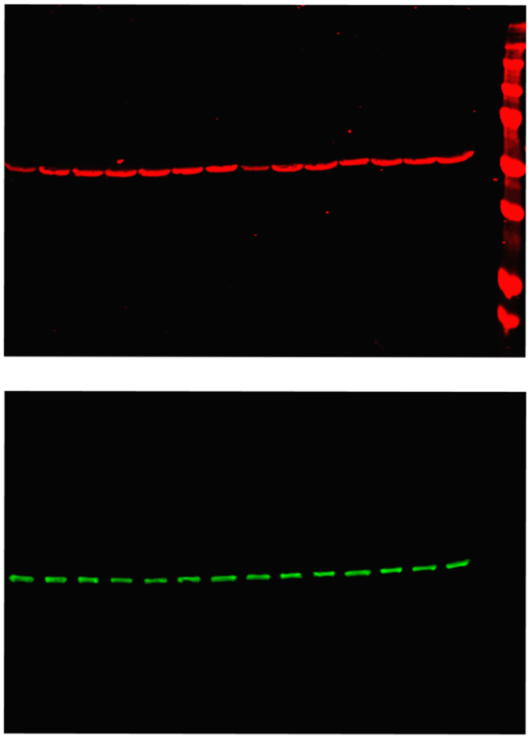

Supp. Fig. S7c

pErk1/2

Actin

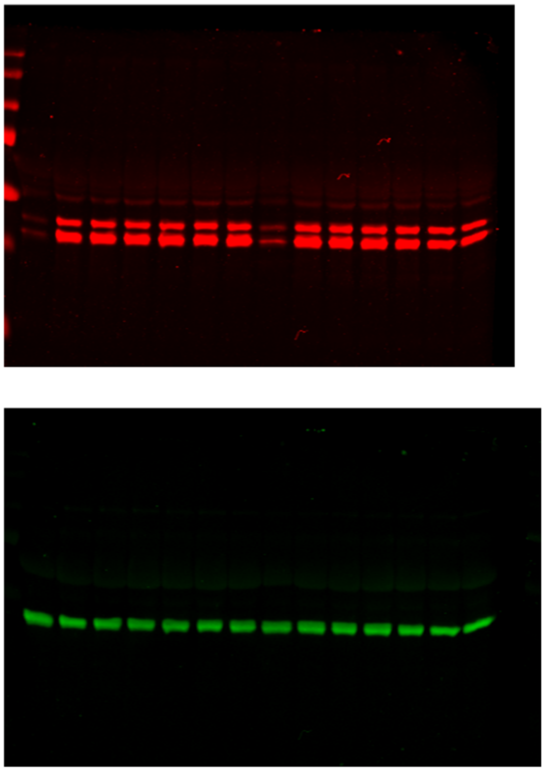

Supplementary Figure 12. Full sized images of immunoblots for Supplementary Figures 5 and 7.

Supplementary Table 1. Antibodies used in this study.

Antibody	Source	Clone	Catalog #	Dilution	Purpose
Rat IgG2a, kappa Isotype Control	BioLegend	RTK2758	400544	1:50	Control for function blocking
Mouse IgG2a	Sigma	UPC-10	M5409	1:200	Control for function blocking
Anti-CD11a	BioXCell	M17/4	BE0006	1:1100	Function blocking
Anti-CD11a APC	BioLegend	M17/4	101119	1:200	Flow cytometry
Anti-CD18	Invitrogen	TS1/18	MA1810	1:50	Function blocking
Anti-GzmB Alexa 647	BioLegend	GB11	515405	1:200	Flow cytometry
Anti-Lamp1 eFluor-660	eBioscience	eBio1D4B	50-1071-82	1:200	Flow cytometry
Anti-CD69 FITC	BioLegend	H1.2F3	104505	1:200	Flow cytometry
Rat IgG2b, kappa Isotype Control	BioLegend	RTK4530	400605	1:200	Flow cytometry
Anti-CD54 FITC	Invitrogen	YN1/1.7.4	11-0541-82	1:200	Flow cytometry
Anti-CD3	BioLegend	145-2C11	100301	1:200	Micropatterning
Anti-CD28	BioXCell	37.51	BE0015-1	1:200	T cell activation
Anti-CD3 biotin	eBioscience	145-2C11	13-0031-81	1:500	Micropatterning
Anti-CD45.2 Fab Alexa Fluor 488	BioLegend	Clone 104	109815	1:200	Traction force microscopy
Anti-talin	Abcam	8D4	157808	1:1000	Western blot
Anti-pAKT Ser 473	Cell Signaling Technology		9271T	1:1000	Western blot
Anti-pErk1/2	Cell Signaling Technology	D13.14.4E	4370T	1:1000	Western blot
Anti-betaActin	Sigma	AC-15	A1978	1:15000	Western blot
Anti-pericentrin	Abcam		ab4448	1:500	Immunocytochemistry
Anti-GAPDH	Cell Signaling Technology	D16H11	5174	1:15000	Western blot
Anti-CD4 BV786	BD Biosciences	RPA-T4	740962	1:200	Flow cytometry
Anti-CD8 BV605	BioLegend	RPA-T8	301040	1:200	Flow cytometry
Anti-GFP	Invitrogen		A11122	1:2000	Western blot
