# Supplementary material for: Mechanically active integrins target lytic secretion at the immune synapse to facilitate cellular cytotoxicity
Source: Nat Commun. 2022 Jun 9;13:3222. doi: 10.1038/s41467-022-30809-3 (PMC9184626; doi:10.1038/s41467-022-30809-3)
Supplement: Supplementary file 3 — Description of Additional Supplementary Information Files [file 41467_2022_30809_MOESM3_ESM.pdf]

Description of Additional Supplementary Files

File name: Supplementary Movie 1.

Description: Control micropatterned surfaces do not elicit T cell activation. OT-1 CTLs loaded with Fura-2-AM were imaged on control micropatterned surfaces containing empty fluorescent streptavidin dots. A representative 200× time-lapse movie is shown. Fura-2 ratio is depicted in pseudocolor with cold and warm colors indicating low and high intracellular Ca^{2+}, respectively. Time in HH:MM:SS is shown in the upper left corner. Scale bar = 10 μm.

File name: Supplementary Movie 2.

Description: T cell activation on stimulatory micropatterned surfaces. OT-1 CTLs loaded with Fura-2-AM were imaged on Dual-spot micropatterned surfaces with both pMHC and ICAM-1 loaded into fluorescent streptavidin dots. A representative 200× time-lapse movie is shown. Fura-2 ratio is depicted in pseudocolor with cold and warm colors indicating low and high intracellular Ca^{2+}, respectively. Time in HH:MM:SS is shown in the upper left corner. Scale bar = 10 μm.

File name: Supplementary Movie 3.

Description: CTL degranulation on a micropatterned surface. OT-1 CTLs expressing pHluorin-Lamp1 were imaged by confocal microscopy on ICAM-spot micropatterned surfaces. A representative 100× time-lapse movie is shown, with pHluorin-Lamp1 and Alexa Fluor 647 streptavidin depicted in cyan and red, respectively. Time in M:SS is shown in the bottom right. Scale bar = 2 μm. The CTL near the center of the field degranulates at 9:46.

File name: Supplementary Movie 4.

Description: CTLs exert pulling forces through the TCR and LFA-1. OT-1 CTLs were imaged on glass substrates coated with pMHC-Atto647N (cyan) and ICAM-1-Cy3B (yellow) MTPs. A representative 143× time-lapse movie is shown, with MTP fluorescence overlaid onto the corresponding IRM image. Time in MM:SS is indicated in the top left corner. Scale bar = 8 μm.

File name: Supplementary Movie 5.

Description: Talin depletion inhibits force exertion through LFA-1, but not the TCR. OT-1 Cas9 CTLs expressing talin specific gRNA (Talin CR) were imaged on glass substrates coated with pMHC-Atto647N and ICAM-1-Cy3B MTPs. Representative 143× time-lapse movies of MTP

fluorescence are shown in montage together with the corresponding IRM signal. Time in MM:SS is indicated in the top left corner of the IRM time-lapse. Scale bar = 8 μm.

File name: Supplementary Movie 6.

Description: Force exertion by talin sufficient CTLs. OT-1 Cas9 CTLs expressing a nontargeting control gRNA (NT CR) were imaged on glass substrates coated with pMHC-Atto647N and ICAM-1-Cy3B MTPs. Representative 143× time-lapse movies of MTP fluorescence are shown in montage together with the corresponding IRM signal. Time in MM:SS is indicated in the top left corner of the IRM time-lapse. Scale bar = 8 μm.
