# Supplementary material for: Mechanically active integrins target lytic secretion at the immune synapse to facilitate cellular cytotoxicity
Source: Nat Commun. 2022 Jun 9;13:3222. doi: 10.1038/s41467-022-30809-3 (PMC9184626; doi:10.1038/s41467-022-30809-3)
Supplement: Supplementary file 10 — Reporting Summary [file 41467_2022_30809_MOESM10_ESM.pdf]

nature portfolio

Corresponding author(s): Morgan Huse

Last updated by author(s): Aug 11, 2021

Reporting Summary

Nature Portfolio wishes to improve the reproducibility of the work that we publish. This form provides structure for consistency and transparency in reporting. For further information on Nature Portfolio policies, see our Editorial Policies and the Editorial Policy Checklist.

Statistics

For all statistical analyses, confirm that the following items are present in the figure legend, table legend, main text, or Methods section.

n/a	Confirmed	
☐	☒	The exact sample size (*n*) for each experimental group/condition, given as a discrete number and unit of measurement
☐	☒	A statement on whether measurements were taken from distinct samples or whether the same sample was measured repeatedly
☐	☒	The statistical test(s) used AND whether they are one- or two-sided<br>*Only common tests should be described solely by name; describe more complex techniques in the Methods section.*
☐	☒	A description of all covariates tested
☐	☒	A description of any assumptions or corrections, such as tests of normality and adjustment for multiple comparisons
☐	☒	A full description of the statistical parameters including central tendency (e.g. means) or other basic estimates (e.g. regression coefficient) AND variation (e.g. standard deviation) or associated estimates of uncertainty (e.g. confidence intervals)
☐	☒	For null hypothesis testing, the test statistic (e.g. F, t, r) with confidence intervals, effect sizes, degrees of freedom and P value noted<br>*Give P values as exact values whenever suitable.*
☒	☐	For Bayesian analysis, information on the choice of priors and Markov chain Monte Carlo settings
☒	☐	For hierarchical and complex designs, identification of the appropriate level for tests and full reporting of outcomes
☒	☐	Estimates of effect sizes (e.g. Cohen's *d*, Pearson's *r*), indicating how they were calculated

Our web collection on statistics for biologists contains articles on many of the points above.

Software and code

Policy information about availability of computer code

Data collection	The following commercially available softwares were used for data collection: Leica Application Suite AF (version 2.7.3.9723), NIS Elements (version 4.30), CytExpert (version 2.3), Metamorph (version 7.8.2.0).
Data analysis	The following commercially available softwares were used for data analysis: Imaris (version 9.5.1), SlideBook (version 6.0.17), Python (version 3.7.10), PRISM (version 9.1.0), Excel (version 16.50), FlowJo (version 10.7.2), ImageJ (version 2.1.0/1.53c), and MATLAB (version 9.9.0.1538559). MTP linescan analysis data and scripts are available on an online GitHub repository (https://github.com/axiezai/degranulation_MTP_fluorescence_average_line_scan_2021).

For manuscripts utilizing custom algorithms or software that are central to the research but not yet described in published literature, software must be made available to editors and reviewers. We strongly encourage code deposition in a community repository (e.g. GitHub). See the Nature Portfolio guidelines for submitting code & software for further information.

Data

Policy information about availability of data

All manuscripts must include a data availability statement. This statement should provide the following information, where applicable:

- Accession codes, unique identifiers, or web links for publicly available datasets
- A description of any restrictions on data availability
- For clinical datasets or third party data, please ensure that the statement adheres to our policy

The data supporting the findings of this study are available within the article and/or its supporting materials. Raw data relevant to the study are available from the corresponding author upon reasonable request.

Field-specific reporting

Please select the one below that is the best fit for your research. If you are not sure, read the appropriate sections before making your selection.

[x] Life sciences   [ ] Behavioural & social sciences   [ ] Ecological, evolutionary & environmental sciences

For a reference copy of the document with all sections, see nature.com/documents/nr-reporting-summary-flat.pdf

Life sciences study design

All studies must disclose on these points even when the disclosure is negative.

Sample size	No statistical methods were used to predetermine sample size. Sample sizes were chosen based on pilot studies and previously published work. Sample size numbers for each experiment can be found in the figure legend.
Data exclusions	No data was excluded from the analyses.
Replication	All experiments were performed at least twice under the same exact conditions to confirm the reproducibility of all experimental findings. All attempts at replication were successful.
Randomization	Samples were not randomly allocated into experimental groups. Most experiments were initiated from the same group of naive T cells or the same group of differentiated CTLs. In studies comparing wild type and LFA knockout T cells, CTLs were differentiated from age and sex-matched wild type and LFA knockout mice.
Blinding	Investigators were not blinded to group allocation, as readouts for flow cytometric and imaging-based assays were evaluated using quantitative metrics that were objectively obvious. Identical analytical protocols were applied to all experimental groups, and every experiment was performed using multiple control samples.

Reporting for specific materials, systems and methods

We require information from authors about some types of materials, experimental systems and methods used in many studies. Here, indicate whether each material, system or method listed is relevant to your study. If you are not sure if a list item applies to your research, read the appropriate section before selecting a response.

Materials & experimental systems

n/a	Involved in the study	
[ ]	[x]	Antibodies
[ ]	[x]	Eukaryotic cell lines
[x]	[ ]	Palaeontology and archaeology
[ ]	[x]	Animals and other organisms
[x]	[ ]	Human research participants
[x]	[ ]	Clinical data
[x]	[ ]	Dual use research of concern

Methods

n/a	Involved in the study	
[x]	[ ]	ChIP-seq
[ ]	[x]	Flow cytometry
[x]	[ ]	MRI-based neuroimaging

Antibodies

Antibodies used	Rat IgG2a, kappa Isotype Control antibody (clone RTK2758, BioLegend, catalog number 400544, final dilution 1:50) was used as an isotype control antibody in co-culture assays.<br>Mouse IgG2a (clone UPC-10, Sigma, catalog number M5409, final dilution 1:200) was used as an isotype control antibody in human co-culture assays.<br>Anti-LFA1 block antibody (clone M17/4, BioXCell, catalog number BE0006, final dilution 1:1,100) was used to inhibit the functional activity of LFA-1 in co- culture assays.<br>APC labeled anti-LFA1 antibody (clone M17/4, Biolegend, catalog number 101119, final dilution 1:200) was used to quantify surface expression of LFA-1.<br>Anti-LFA1 block antibody (clone TS1/18, Invitrogen, catalog number MA1810, final dilution 1:50) was used to inhibit the functional activity of human LFA-1 in co-culture assays.<br>Alexa 647 labeled anti-GzmB antibody (clone GB11, Biolegend, catalog number 515405, final dilution 1:200) was used to stain for intracellular GzmB levels via flow cytometry.<br>eFluor 660–labeled anti-Lamp1 antibody (clone eBio1D4B, eBioscience, catalog number 50-1071-82, final dilution 1:200) was used to measure surface exposure of Lamp-1 in response to antigen via flow cytometry.<br>FITC labeled anti-CD69 antibody (clone H1.2F3, BioLegend, catalog number 104505, final dilution 1:200) was used to measure upregulation of CD69 in response to antigen via flow cytometry.<br>Rat IgG2b, kappa Isotype Control Antibody (clone RTK4530, BioLegend, catalog number 400605, final dilution 1:200) was used as an isotype control antibody for detection of cell surface CD54 (ICAM-1) via flow cytometry.

FITC labeled anti-CD54 antibody (clone YN1/1.7.4, Invitrogen, catalog number 11-0541-82, final dilution 1:200) was used to detect cell surface expression of CD54 (ICAM-1) via flow cytometry.
Anti-CD3 antibody (clone 145-2C11, BioLegend, catalog number 100301, final dilution 1:200) was used to coat inter-stamp space for micropatterning experiments and activate polyclonal T cells.
Anti-CD28 antibody (clone 37.51, BioXcell, catalog number BE0015-1, final dilution 1:200) was used to activate polyclonal T cells.
Biotinylated anti-CD3 antibody (clone 145-2C11, eBioscience, catalog number 13-0031-81, final dilution 1:500) was used to coat stamped streptavidin for micropatterning experiments.
Anti-CD45.2 Fab Alexa Fluor 488 antibody (clone 104, BioLegend, catalog number 109815, final dilution 1:200) was used to generally label the T cell surface for traction force microscopy on micropillars.
Anti-talin antibody (clone 8D4, Abcam, catalog number 157808, , final dilution 1:1000) was used to confirm the knockout of talin in response to CRISPR/ Cas9-mediated gene knockout via immunoblot.
Anti-pAkt antibody (Phospho-Akt (Ser473) antibody, Cell Signaling Technology, catalog number 9271T, final dilution 1:1,000) was used to detect the phosphorylation of Ser473 in Akt in response to antigen via immunoblot.
Anti-pErk1/2 antibody (clone D13.14.4E, Phospho-Thr202/Tyr204, Cell Signaling Technology, catalog number 4370T, final dilution 1:1,000) was used to detected the phosphorylation of Thr202 and Tyr204 in Erk1/2 in response to antigen via immunoblot.
Anti-beta-actin antibody (clone AC-15, Sigma-Aldrich, catalog number A1978, final dilution 1:15,000) was used as a loading control for immunoblot.
Anti-pericentrin antibody (clone number not provided, Abcam, catalog number ab4448, final dilution 1:500) was used to detect the centrosome in immunostaining experiments.
Anti-GAPDH antibody (clone D16H11, Cell Signaling, catalog number 5174, final dilution 1:15,000) was used as a loading control for immunoblot.
BV786 labeled anti-CD4 antibody (clone RPA-T4, BD Biosciences, catalog number 740962, final dilution 1:200) was used to detect human CD4.
BV605 labeled anti-CD8 antibody (clone RPA-T8, BioLegend, catalog number 301040, final dilution 1:200) was used to detect human CD8.
Anti-GFP polyclonal antibody (Invitrogen, catalog number A11122, final dilution 1:2000) was used to detect Talin head domain GFP fusion protein by Western blot.

Validation

All antibodies were commercially validated using company-specific methods, and then validated in-house against a positive control cell sample.

BioLegend statement: (https://www.biolegend.com/en-us/quality-control)
The specificity and sensitivity of each antibody is thoroughly validated in the New Product Development stage. This is done by staining multiple target cells with either single- or multi-color analysis or by other testing approaches. The QC specifications and testing SOPs and gold standard for each product are then developed.

Sigma statement: https://www.sigmaaldrich.com/US/en/technical-documents/technical-article/protein-biology/immunohistochemistry/antibody-enhanced-validation
It is common for commercial antibody developers to assess the on-target binding of an antibody during post immunization screening solely by Western blot (WB) or immunohistochemistry (IHC). This screen ensures that the antibody in development is, at this initial stage, recognizing the expected target and performing similarly to previous lots, if applicable. However, this simple screening is not adequate to assure application suitability and true lot-to-lot consistency. We therefore subsequently test in as many additional immunodetection applications as practical in samples chosen to be relevant to the intended use of the product. These include immunohistochemistry, immunocytochemistry (ICC), Western blot, ELISA, immunoprecipitation, and more. This in-depth application testing can help assess the antibody's specificity for the target and provides contextually relevant validation in applications and samples most likely to be used by our customers. This application-specific data should be reviewed by the researcher and appropriately assessed for the researchers intended use. Beyond review of the vendor generated application data, critical review of the epitope, species reactivity, clonality, appropriate host species, and development of appropriate controls are critical responsibilities of the researcher in the selection and use of antibody product.

BioXCell statement: https://bxcell.com/the-bio-x-cell-advantage/
We utilize a library of recombinant proteins and our bioassay expertise to validate that each lot of applicable InVivoPlus antibody binds strongly and specifically to its target antigen.

ThermoFisher (Invitrogen, eBioscience) statement: (https://www.thermofisher.com/us/en/home/life-science/antibodies/invitrogen-antibody-validation.html).
Thermo Fisher Scientific is committed to adopting validation standards for our Invitrogen antibody portfolio that are tested for both target specificity and functional application. By supporting the International Working Group for Antibody Validation (IWGAV), and adopting their recommendations, we're doing our part to ensure reproducibility and proper functionality in the scientific community.

Abcam statement: https://www.abcam.com/primary-antibodies/how-we-validate-our-antibodies
Antibodies are validated in western blot using lysates from cells or tissues that we have identified to express the protein of interest. Once we have determined the right lysates to use, western blots are run and the band size is checked for the expected molecular weight. We will always run several controls in the same western blot experiment, including positive lysate and negative lysate (if possible, Figure 2). When possible, we also include knock-out (KO) cell lines as a true negative control for our western blots. We are always increasing the number of KO-validated antibodies we provide. In addition, we run old stock alongside our new stock. If we know the old stock works well, this also acts as a suitable positive control. If the western blot result gives a clear clean band and we are happy with the result from the control lanes, these antibodies will be passed and added to the catalog.
IHC and ICC determine whether an antibody recognizes the correct protein based on cellular and subcellular localization. Antibody specificity is confirmed by looking at cells that either do or do not express the target protein within the same tissue. Initially, our

scientists will review the available literature to determine the best cell lines and tissues to use for validation. We then check the protein expression by IHC/ICC to see if it has the expected cellular localization (Figure 3). If the localization of the signal is as expected, this antibody will pass and is considered suitable for use in IHC/ICC. We use a variety of methods, including staining multi-normal human tissue microarrays (TMAs), multi-tumor human TMAs, and rat or mouse TMAs during antibody development. These high-throughput arrays allow us to check many tissues at the same time, providing uniformly as all tissues are exposed to the exact same conditions. We are currently working towards using KO cell lines for our ICC validation.

Cell Signaling statement: https://www.cellsignal.com/about-us/cst-antibody-validation-principles
At Cell Signaling Technology (CST), we understand that there is no single assay that can determine the validity of an antibody. Confirming that an immunoreagent is sufficiently specific and sensitive depends on the application and protocol being used, the type and quality of sample being analyzed, and the inherent biophysical properties of the antibody itself. To ensure our antibodies will work in your experiment, we adhere to the Hallmarks of Antibody Validation™, six complementary strategies that can be used to determine the functionality, specificity, and sensitivity of an antibody in any given assay. CST adapted the work by Uhlen, et. al., ("A Proposal for Validation of Antibodies." Nature Methods (2016)) to build the Hallmarks of Antibody Validation, based on our decades of experience as an antibody manufacturer and our dedication to reproducible science.

BD Biosciences statment: https://www.bdbiosciences.com/en-us/products/reagents/flow-cytometry-reagents/research-reagents/quality-and-reproducibility
We understand the value of consistency, and that's reflected in the rigorous testing and quality control standards we place on our products. In particular, we recognize the importance of reagent lot-to-lot consistency; thus, we continue to strive for the highest quality measures to help meet the data reproducibility needs of your research.

Eukaryotic cell lines

Policy information about cell lines

Cell line source(s)	RMA-s cell line were a gift from Gregoire Altan-Bonnet (NIH). Phoenix-ECO cells was purchased from ATCC. The B16F10 cell line was provided by Jedd Wolchok (MSKCC).
Authentication	Cell morphology and surface level expression of key molecules was assessed to validate cell identity.
Mycoplasma contamination	Cell lines were not tested for mycoplasma contamination.
Commonly misidentified lines (See ICLAC register)	No commonly misidentified cell lines were used.

Animals and other organisms

Policy information about studies involving animals; ARRIVE guidelines recommended for reporting animal research

Laboratory animals	OT-1 TCR transgenic mice (Jackson Labs #003831); Cas9 knockin mice (Jackson Labs #026179); cd11a-/- mice (Jackson Labs #042053); C57BL/6 mice (Jackson Labs #000664); and cd11a-yfp knockin mice (gift from Minsoo Kim, University of Rochester)<br>Mice of both sexes were euthanized for lymphocyte extraction between 6 and 12 weeks of age.
Wild animals	No wild animals were used for this study.
Field-collected samples	No field-collected samples were used for this study.
Ethics oversight	The animal protocols used for this study were approved by the Institutional Animal Care and Use Committee of Memorial Sloan Kettering Cancer Center.

Note that full information on the approval of the study protocol must also be provided in the manuscript.

Flow Cytometry

Plots

Confirm that:

- [x] The axis labels state the marker and fluorochrome used (e.g. CD4-FITC).
- [x] The axis scales are clearly visible. Include numbers along axes only for bottom left plot of group (a 'group' is an analysis of identical markers).
- [x] All plots are contour plots with outliers or pseudocolor plots.
- [x] A numerical value for number of cells or percentage (with statistics) is provided.

Methodology

Sample preparation	Mature OT-I CTLs were obtained as described in the methods section. Cells were pelleted and washed twice in FACS buffer (PBS with 2% FBS, 1mM EDTA, 0.02% azide) before being stained on ice with experiment-relevant antibodies for 20 min. Cells were then washed again using FACS buffer for sample collection via flow cytometry.

Instrument	The Beckman Coulter CytoFLEX LX flow cytometer was used to collect flow cytometry data in this study.
Software	CytExpert software(version 2.3) was used to collect flow cytometry data. FlowJo software (version 10.7.2) was used to analyze flow cytometry data.
Cell population abundance	No cells were sorted for this study.
Gating strategy	Co-culture populations were stained with CellTrace-Violet or PKH26 and gated with at least one log-fold difference in fluorescence between them. Live CTLs were gated according to standard FSC/SSC gates.

☒ Tick this box to confirm that a figure exemplifying the gating strategy is provided in the Supplementary Information.
